# The therapeutic potential of resveratrol: a review of clinical trials

**DOI:** 10.1038/s41698-017-0038-6

**Published:** 2017-09-25

**Authors:** Adi Y. Berman, Rachel A. Motechin, Maia Y. Wiesenfeld, Marina K. Holz

**Affiliations:** 1 Department of Biology, Yeshiva University, New York, NY USA; 2 Department of Molecular Pharmacology and the Albert Einstein Cancer Center, Albert Einstein College of Medicine, Bronx, NY USA

## Abstract

Resveratrol is a nutraceutical with several therapeutic effects. It has been shown to mimic effects of caloric restriction, exert anti-inflammatory and anti-oxidative effects, and affect the initiation and progression of many diseases through several mechanisms. While there is a wealth of in vitro and in vivo evidence that resveratrol could be a promising therapeutic agent, clinical trials must confirm its potential. In this work, we reviewed the current clinical data available regarding the pharmacological action of resveratrol. Most of the clinical trials of resveratrol have focused on cancer, neurological disorders, cardiovascular diseases, diabetes, non-alcoholic fatty liver disease (NAFLD), and obesity. We found that for neurological disorders, cardiovascular diseases, and diabetes, the current clinical trials show that resveratrol was well tolerated and beneficially influenced disease biomarkers. However resveratrol had ambiguous and sometimes even detrimental effects in certain types of cancers and in NAFLD. In most of the clinical trials, the major obstacle presented was resveratrol’s poor bioavailability. Thus, this work provides useful considerations for the planning and design of future pre-clinical and clinical research on resveratrol.

## Introduction

Resveratrol (3,4′,5- trihydroxystilbene) is a nutraceutical that has recently attracted a lot of research attention due to its exciting pharmacological potential. It is a phytoalexin found in many plants including grapes, peanuts, and berries. Resveratrol was first isolated in *Veratrum grandiflorum*, or white hellebore plant, in the 1940’s.^1^ Stilbene compounds are known for their ability to provide plants with resistance to microbial and fungal infection,^2^ and early research showed that resveratrol was present in large quantities in injured, infected, and ultraviolet-treated leaves.^3^ Processed plants products also contain a significant amount of resveratrol; its presence in red wine (concentrations of 0.1–14.3 mg/L) has been suggested as a solution to the “French paradox,” the observation of an unexpectedly low rate of heart disease among Southern French people who consume a lot of red wine, despite their diets being high in saturated fat.^4–6^

Resveratrol is an activator of SIRT1, one of the mammalian forms of the sirtuin family of proteins.^7^ SIRT1 deacetylates histones and nonhistone proteins including transcription factors. The SIRT1-regulated pathway affects metabolism, stress resistance, cell survival, cellular senescence, inflammation-immune function, endothelial functions, and circadian rhythms. Resveratrol has been shown to activate SIRT1 and therefore is predicted to benefit diseases affected by abnormal metabolic control, inflammation, and cell cycle defects.

As a natural compound, resveratrol’s use as a nutraceutical and as a therapeutic agent for many diseases has been widely researched in preclinical studies. Its use is especially of interest for cancer patients because of the high risks associated with traditional treatments, including surgery and chemotherapy. The complexities in cancer cell signaling networks obstruct the therapeutic success of specific inhibitors that target only one network. However, because resveratrol has been shown in vitro and in vivo to have chemopreventive and chemotherapeutic effects on cancers by targeting multiple pathways, it is a promising anticancer agent.^8^ Resveratrol affects all three stages of carcinogenesis: initiation, promotion, and progression. Furthermore, resveratrol has been shown to directly induce the apoptotic pathway through several mechanisms.^9–11^

For example, resveratrol affects the nuclear factor κB (NF-κB) signaling pathway which regulates inflammation, immune response to infection, and cellular response to stimuli.^12^ In addition, it has been shown to significantly inhibit the IGF-1R/Akt/Wnt pathways and activate p53, and therefore can influence cell development and tumorigenesis.^13^ Furthermore, resveratrol can inhibit the phosphatidylinositol 3-kinase (PI3K)/Akt pathway to regulate cell differentiation, growth, proliferation, and several other activities.^14–16^ Several studies have uncovered the various mechanisms through which resveratrol acts on the PI3K/Akt pathway. For instance, our lab has shown that resveratrol inhibits Akt signaling in multiple cancer cells exhibiting hyperactivated PI3K/Akt/mechanistic target of rapamycin (mTOR) signaling, and therefore might be a useful therapy when used in combination with other PI3K/Akt/mTOR inhibitors.^17–22^

The NF-κB, Wnt, and PI3K/Akt/mTOR pathways are also involved in several other diseases, such as various metabolic, neurodegenerative, and cardiovascular disorders. By acting on these pathways and others, resveratrol has been shown to be a promising therapeutic agent for such diseases. Resveratrol also inhibits cyclooxygenases (COX), which are responsible for the conversion of arachidonic acid into prostaglandins.^12^ The suppression of this pathway reduces inflammation and suggests the possibility of resveratrol as a treatment for inflammatory conditions.

Resveratrol has also been found to inhibit the expression of vascular cell adhesion molecules (VCAM) and to affect the activity of vascular smooth muscle cells which are responsible for the development of atherosclerosis and hypertension, respectively.^23^ Additionally, studies have shown that resveratrol inhibits platelet aggregation and activation in vitro, which may be effective in preventing blood clot formation and ultimately myocardial infarction and stroke.^23^

From a pharmacokinetic perspective, resveratrol has proven to be more effective when applied topically rather than administered orally because it is quickly metabolized and excreted.^24^ This poor bioavailability can be attributed to the rapid conjugation of trans-resveratrol to glucuronic acid and sulfates, producing glucuronides and sulfate conjugates which accumulate in plasma and urine.^25^ Many trials are in progress to determine the appropriate oral dosage given the rapid metabolism of resveratrol, and to study resveratrol derivatives such as SRT501, a micronized oral version of resveratrol that may have higher bioavailability.^26,27^ A recent study demonstrated nonlinear dose response effects of resveratrol’s activity, underscoring the need to consider the intricate and complex issue of low dose (diet related) versus high dose (pharmacological) differences of resveratrol responses.^28^

**Fig. 1:**
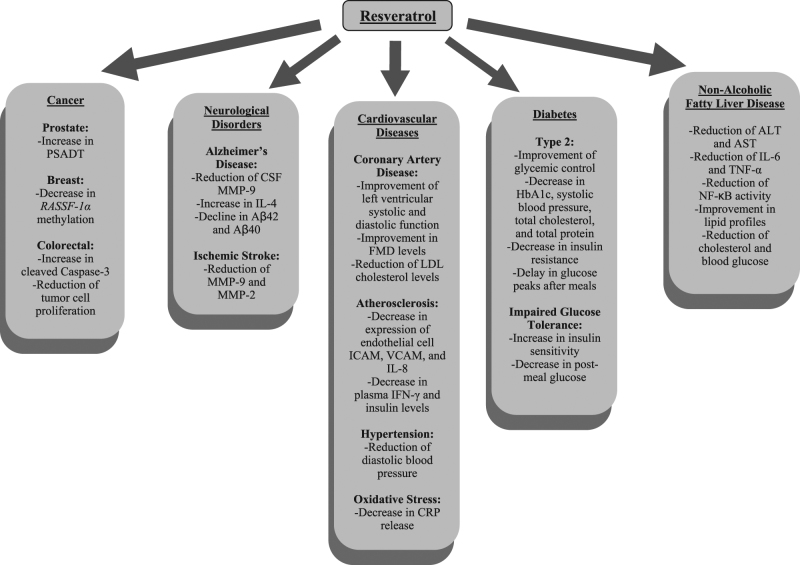
Clinical benefits of resveratrol

Despite an abundance of laboratory and animal research, there is little clinical evidence that resveratrol is an effective therapeutic in humans.^29^ We have therefore compiled the clinical data that have been accumulated thus far in order to examine resveratrol’s efficacy in cancer, neurological disorders, cardiovascular diseases, diabetes, NAFLD, and obesity. These disease categories were chosen because most of the clinical data available for resveratrol pertains to them. The aspects of resveratrol studied in these clinical trials range from its pharmacokinetics and pharmacodynamics, its effect on essential biomarkers for these diseases, to any adverse events that occurred as a result of resveratrol treatment. A summary of the clinical findings for each disease is presented in Table 1, and Fig. 1 depicts a summary of the clinical benefits of resveratrol found in the clinical trials presented here. Our aim is to assist the research community by providing a summary of the current clinical knowledge available on resveratrol so that future studies can properly orient their goals.

**Table 1:** Summary of Resveratrol’s clinical effects

Disease type	Study conditions	Length of trial	Resveratrol dosage	Biomarker changes	Effect	Reference
Cancer						
Prostate cancer	14 patients, phase 1 trial	2–31 months (depending on patient)	500, 1000, 2000, 3000, or 4000 mg of MPX. Every 500 mg MPX has 4.4 μg resveratrol	Increase in PSADT	Beneficial	^33^
Prostate cancer	66 patients, randomized, placebo-controlled, single-site clinical trial	4 months	150 mg or 1000 mg daily	Decrease in androstenedione, DHEA, and DHEAS. No effect on prostate size and PSA levels	None	^34^
Colorectal cancer	9 patients randomized, placebo- controlled, double blind, phase 1 trial	14 days prior to surgery	5.0 g SRT501	Increase in cleaved Caspase-3 (apoptosis)	Beneficial	^26^
Colorectal cancer	20 patients	8 days prior to surgery	500 or 1000 mg	Reduction in tumor cell proliferation, indicated by reduction in Ki-67 staining	Beneficial	^37^
Multiple myeloma	24 patients, phase 2 trial	~4 months	5.0 g SRT501	NA	Severe adverse events	^27^
Breast cancer	39 patients, randomized, double-blind, placebo- controlled clinical trial	3 months	5 or 50 mg twice daily	Decrease in *RASSF-1α* methylation	Beneficial	^39^
Neurological disorders						
AD	119 patients, randomized, placebo- controlled, double blind, multi-site, phase 2 trial	12 months	500 mg once daily, with 500 mg dose escalation every 13 weeks, ending with 1000 mg twice daily	Reduced CSF MMP9, increase IL-4, attenuated decline in Aβ42 and Aβ40	Beneficial	^43^
AD	119 patients, randomized, placebo-controlled, double-blind, multicenter, phase 2 trial	12 months	500 mg once daily, with 500 mg dose escalation every 13 weeks, ending with 1000 mg twice daily	Attenuated decline in Aβ42 and Aβ40 increased brain volume loss	Beneficial	^44^
Ischemic stroke	312 patients, randomized, placebo-controlled	60 min after 0–2 h of stroke onset	2.5 mg resveratrol/kg of body weight	Reduced MMP-9 and MMP-2	Beneficial	^48^
Cardiovascular diseases						
Coronary artery disease	40 patients, double-blind, randomized, placebo-controlled	3 months	10 mg daily	Improved left ventricular systolic and diastolic function; improved FMD; lowered LDL-cholesterol level	Beneficial	^52^
Atherosclerosis	44 healthy subjects, double-blind, randomized, placebo-controlled	1 month	400 mg trans-resveratrol, 400 mg grapeskin extract, 100 mg quercetin	Decreased expression of endothelial cell ICAM, VCAM and IL-8; decreased levels of plasma IFN-γ and insulin	Beneficial	^53^
Hypertension	18 patients, double-blind, randomized, placebo-controlled, crossover design	28 days	330 mg grape seed and skin, 100 mg green tea, 60 mg resveratrol, 60 mg blend of quercetin, ginkgo biloba and bilberry	Reduced diastolic pressure	Beneficial	^56^
Inflammation and oxidative stress	50 healthy adult smokers, double-blind, randomized, crossover design	3 months	500 mg daily	Reduced systemic inflammation in airways, decreased CRP release from the liver	Beneficial	^57^
Serum glucose and cardiovascular risk factors	19 schizophrenic male patients, double-blind, randomized, controlled	1 month	200 mg daily	No change in body weight, waist circumference, glucose, and total cholesterol	None	^58^
Cardiovascular health of overweight and obese subjects	45 overweight and slightly obese subjects, randomized, placebo-controlled, crossover design	1 month	150 mg daily	No change in apoA-I concentrations and HDL levels	None	^59^
Diabetes						
Type 2 diabetes	14 patients, double-blind, randomized, placebo-controlled, crossover design	25-week intervention periods with 5-week washout period in between	500 mg twice daily	No effect on GLP-1 secretion	None	^62^
Type 2 diabetes, glycemia	62 patients, prospective, open-label, randomized, controlled trial	3 months	250 mg daily	Improved glycemic control: decreased HbA1c, systolic BP, total cholesterol, and total protein	Beneficial	^63^
Type 2 diabetes	19 patients, double-blind, randomized, placebo-controlled study	1 month	5 mg twice daily	Decreased insulin resistance, decreased blood glucose, delayed glucose peaks after meals, urinary ortho-tyrosine excretion	Beneficial	^64^
IGT	10 patients with mean age 72 ± 3 years, open-label study	1 month	1000, 1500, or 2000 mg daily	Decrease in peak postmeal glucose and 3-h glucose, increased insulin sensitivity	Beneficial	^65^
NAFLD						
NAFLD	28 patients, randomized, placebo- controlled	6 months	1500 mg daily	No change in ALT No improvement in lipid profile or insulin sensitivity	None	^67^
NAFLD	20 patients, randomized, placebo- controlled	2 months	3000 mg	No change in insulin resistance or steatosis. Increase in ALT and AST	None	^69^
NAFLD	60 patients, randomized, placebo- controlled, double blind	3 months	300 mg twice daily	Reduced AST, ALT, cholesterol, glucose, TNF-α	Beneficial	^70^
NAFLD	50 patients, randomized, double-blind, placebo- controlled	3 months	500 mg (in addition to exercise and healthy diet)	Reduction in ALT, IL-6, NF-κB activity improved lipid profiles	Beneficial	^71^

## Clinical trials

### Cancer

A simple search of “resveratrol and cancer” on PubMed yields over 2600 results as of March 2017, while limiting the search to show only clinical trials generates only 15 results, with several of them focusing solely on the general pharmacokinetics of resveratrol. The scarcity of clinical evidence brings into question resveratrol’s viability as an anti-cancer therapeutic. In vitro and in vivo studies show that resveratrol targets COXs, which generate pro-inflammatory molecules that lead to tumor proliferation, and downregulates the AKT, MAPK, and NF-κB signaling pathways, all of which would reduce inflammation and prevent tumorigenesis.^22,30–32^ Clinical studies, however, must determine if the same effects can be seen in human patients. The few clinical trials that have been conducted show that resveratrol has several targets within the cell, and its efficacy is dependent on the type and stage of cancer, dosage levels, and treatment periods.

Even within clinical studies focusing on the same type of cancer, the conclusions drawn can be conflicting or ambiguous. For example, one study done on prostate cancer pathogenesis showed that resveratrol could potentially be used to delay cancer recurrence. Around 33–50% of prostate cancer patients experience biochemical recurrence of the disease after primary treatment. Rising levels of prostate-specific antigen (PSA) are the earliest indication of disease recurrence. MPX, pulverized muscadine grape skin which contains resveratrol, delayed the development of recurrence by lengthening the PSA doubling time (PSADT) by 5.3 months. However, these results were not statistically significant.^33^ This study is continuing in the phase 2 portion of the clinical trial, so it remains to be seen if MPX is a viable treatment option. In contrast, a second clinical trial involving prostate cancer and resveratrol definitively concluded that it would not be a viable treatment. Despite pre-clinical evidence that resveratrol reduces androgen production and modulates androgen receptor activity, Kjaer et al. concluded that resveratrol could not treat prostate cancer as it had no effect on prostate volume or PSA levels.^34^ From these studies, it seems unlikely that resveratrol will prove to be an effective treatment for prostate cancer, but more clinical trials need to be performed to confirm this.

Similarly, in clinical trials conducted in patients with colorectal cancer, the results seem promising, but remain inconclusive regarding whether or not resveratrol could be a viable treatment. In vitro studies showed that resveratrol inhibits growth and induces apoptosis in human colon cells, and murine models of colorectal cancer showed that resveratrol inhibited colorectal inflammation and carcinogenesis.^35,36^ Therefore, two clinical trials aimed to determine the pharmacokinetics of resveratrol in colorectal tissue or hepatic metastases. After around 1–2 weeks of treatment with resveratrol or SRT501, the measured concentrations of parent resveratrol and its major metabolites in the colorectal tissue of patients were similar to the effective concentrations of resveratrol used in preclinical studies. However, the anticarcinogenic activity of resveratrol’s metabolites has yet to be experimentally verified, so it is unclear whether this finding provides additional justification for pursuing resveratrol as a treatment for colon cancer. Additionally, the levels of Ki-67, a proliferation marker, and caspase-3, an apoptotic marker, were affected only slightly in tissue samples.^26,37^ While this proves that resveratrol has some pharmacological effect, it is unclear if this effect is significant enough to make it a useful therapeutic agent for colon cancer.

There are other studies though where the potential of resveratrol as a treatment seems more tenuous, such as in certain types of multiple myeloma. Resveratrol was found to inhibit NF-κB, AKT, and STAT3 and exhibit cytotoxicity in multiple myeloma cell lines.^38^ SRT501 was tested in patients with relapsed or refractory multiple myeloma. Despite convincing pre-clinical evidence that resveratrol could aid in the treatment of patients with multiple myeloma, the clinical trial found that it caused several severe adverse events, the most prominent of which was renal failure.^27^ As SRT501 was proven to be safe in a phase 2 clinical trial, and it did not cause any nephrotoxicity in a phase 1 study for colorectal cancer patients, this adverse event seemed to be specific to multiple myeloma patients. These results indicate that SRT501 and likely any other versions of resveratrol could not be a possible treatment for multiple myeloma.

Alternatively, in breast cancer, one clinical trial encouragingly showed that resveratrol was safe and well tolerated, albeit the length of treatment was considerably shorter than that in the multiple myeloma study. Participants were treated with resveratrol for 12 weeks. The study found that as time progressed, increasing amounts of resveratrol were detected in blood serum samples. Furthermore, the trial also revealed that the drug affected the epigenetic pattern of *RASSF-1α*, a gene associated with breast cancer, and this effect correlated with the levels of circulating resveratrol.^39^ These results suggest that resveratrol may act as a chemopreventive agent for breast cancer by influencing the epigenetics of breast cancer associated genes, a finding that needs to be confirmed in future clinical trials.

From these clinical trials, it is clear that there is still much to learn about the use of resveratrol as a cancer therapeutic. It seems that resveratrol may have specificity for certain types of cancers, but even this is difficult to definitively conclude given the small number of clinical trials that have been conducted. In contrast, extensive research has been conducted on the chemotherapeutic activity of resveratrol in in vitro and in vivo models, and many of these findings implicate the potential utility of resveratrol in a clinical setting. For example, our group has found resveratrol to be a potent complement to mTOR inhibitors in diseases affected by mTOR hyperactivation.^22^ While rapamycin has been shown to be a viable therapeutic agent in certain cancers, it is ineffective in monotherapy due to the regulatory feedback loop between mTOR and Akt. When mTOR is active, it represses Akt activity. Therefore, the inhibition of mTOR by rapamycin allows the tumor cells to survive through reactivation of the pro-survival Akt signaling. Our group has shown that resveratrol has the ability to inhibit Akt signaling and induce apoptosis. Therefore, when used in combination with rapamycin, resveratrol elicited cytotoxicity in cells. This effect was seen in bladder and breast cancer cell lines, lymphangioleiomyomatosis-derived cells, tuberous sclerosis complex 2 (TSC2)-deficient cell lines, as well as in TSC2-deficient mouse xenograft tumors.^17,19–21^

These and many other studies justify the need for more clinical trials studying resveratrol through its various mechanisms of action. Additionally, the metabolites of resveratrol should be studied as well, since some of them have been shown to have anticancer activities.^40^ Based on the clinical trials presented here, it seems that the primary obstacles that need to be overcome before resveratrol can be used as a cancer therapeutic are its bioavailability and related adverse events. There have been several reviews written on the topic of resveratrol’s poor bioavailability, highlighting the need to focus efforts on improving the pharmaceutical properties of resveratrol.^24^ This need is underscored by the vast amount of preclinical studies that provide promising evidence for the use of resveratrol as a cancer therapeutic agent.

### Neurological disorders

Neurological disorders such as Alzheimer’s disease (AD) and stroke are thought to occur via oxidative and inflammatory damage to the central nervous system (CNS). As resveratrol has been shown to have strong anti-inflammatory and anti-oxidative effects, many have hypothesized that resveratrol could be a useful potential treatment for neurological disorders. Additionally, resveratrol is known to modulate the activities of AMPK, SIRT1, and PGC-1α, metabolic regulators which have been shown to be involved in the onset of neurological disorders.^41^

While the exact mechanism of AD development is unknown, several biomarkers have been identified which help to characterize disease onset and progression and may serve as therapeutic targets. For instance, amyloid-β (Aβ) plaque accumulation caused by amyloid beta precursor protein (APP) and apoliporprotein E (APOE) genetic mutations and increased inflammation and oxidative damage have been shown to be associated with AD.^42^ Additionally, caloric restriction has been shown to prevent onset of neurological disorders.^41^ Caloric restriction activates the deacetylases sirtuins, most notably SIRT1, which is also activated by resveratrol.^7^

While clinical studies on the effects of resveratrol in AD remain scarce, two phase 2 clinical trials have shown that resveratrol is safe to use in patients with mild to moderate AD, and that it alters several AD biomarkers. In trials performed by Muossa et al. and Turner et al., patients were treated with resveratrol for 1 year.^43,44^ As compared to patients in the placebo group, patients treated with resveratrol were found to have decreased levels of MMP-9, a matrix metalloproteinase (MMP) that degrades components of the extracellular matrix, an activity that is associated with AD/neurodegeneration. The decrease in MMP-9 may indicate that resveratrol fortifies the CNS by reducing permeability, and thus reducing the ability of pro-inflammatory agents from reaching the brain. Furthermore, patients receiving resveratrol had a slower decline of cerebrospinal fluid (CSF) beta amyloid (Aβ) 42 and Aβ40 levels, indicating lower accumulation of Aβs in the brain.^45^ While resveratrol was shown to be metabolized quickly, limiting its bioavailability, an issue observed in several resveratrol trials, significant amounts of resveratrol and its metabolites were found in the CSF, indicating its ability to successfully cross the blood–brain barrier (BBB).^43,44^ Both clinical trials presented results that were in line with results found in in vitro and in vivo studies, and provided evidence that resveratrol could be a safe and effective treatment for AD.

Similar to AD, MMPs were found to be important biomarkers in brain ischemic stroke, a neurological disorder that accounts for the highest levels of morbidity and mortality worldwide.^46^ Recombinant tissue plasminogen activator (r-tPA) is currently the only safe and effective treatment for brain ischemia.^47^ However, its therapeutic window is severely limited, and it must be administered within 3 h of stroke onset. Delayed r-tPA treatment has been shown to cause up-regulation of MMPs. As shown in the AD resveratrol trials, resveratrol is capable of decreasing the levels of MMP-9, and thus reduces the permeability of the CNS and strengthened the BBB. In a trial conducted by Chen et al., patients who had suffered from a stroke with a clearly defined time of onset were treated with resveratrol along with r-tPA treatment.^48^ The study showed that resveratrol improved the outcome for patients receiving delayed r-tPA treatment. Additionally, a correlation was determined between reduced levels of MMP-9 and MMP-2 and improved treatment outcomes. Thus, by attenuating the up-regulation of MMPs, resveratrol extends the therapeutic window of r-tPA, providing a more efficient treatment for those affected by brain ischemic stroke.

These clinical trials suggest that resveratrol is effective in reducing biomarkers associated with AD and brain ischemic stroke, and exhibited sufficient bioavailability at the dosages used without any extreme adverse events. Thus they provide a rationale for further testing of resveratrol in future clinical trials, and for studying its effectiveness in treating other neurological disorders.

### Cardiovascular diseases

Cardiovascular diseases currently cause over 610,000 deaths each year in the United States. The World Health Organization (WHO) reports that globally, more people die each year from cardiovascular diseases than any other cause.^49^ Because of its anti-inflammatory and anti-oxidative properties, resveratrol has been suggested to promote cardiovascular health, and has therefore been extensively studied as a treatment for prevalent cardiovascular diseases.^50^

Resveratrol has been shown to affect multiple molecular targets that are associated with cardioprotective effects.^51^ For example, resveratrol has been shown to promote endothelial function which may help prevent atherosclerosis and coronary artery disease. A study performed by Magyar et al. found that treatment with resveratrol improved left ventricular systolic and diastolic function. Additionally, resveratrol improved flow-mediated dilation (FMD), which when impaired causes endothelial dysfunction. The treatment also inhibited platelet aggregation and decreased low density lipoprotein (LDL) cholesterol levels.^52^ These results suggest that resveratrol may be useful in treating coronary artery disease. Development of atherosclerosis is also characterized by lipid deposition in arteries, causing blockages and thrombus formation. Treatment with resveratrol decreases the expression of intercellular adhesion molecules (ICAM), vasuclar cell adhesion molecules (VCAMs), and interleukin (IL)-8 molecules which contribute to the development of atherosclerosis by promoting lipid deposition and increased inflammation. Further, resveratrol has been shown to reduce inflammatory biomarkers in patients who do not yet have a high risk of developing atherosclerosis, indicating that resveratrol may be a promising prophylactic treatment.^53^ A one-year trial in patients at high risk of cardiovascular disease showed that consumption of a grape nutraceutical containing resveratrol improved the inflammatory and fibrinolytic status.^54,55^ These anti-inflammatory effects reduce the incidence of atherosclerosis and may therefore decrease the risk of coronary artery disease and stroke.

A study performed by Biesinger et al. determined that resveratrol effectively reduces diastolic blood pressure in conjunction with other phytochemicals such as grape seed extract, green tea supplements and quercetin.^56^ Resveratrol, therefore, may be efficacious in treating patients with hypertension, a major cause of heart disease.

Furthermore, a clinical trial was conducted to determine the effects of resveratrol on the cardiovascular health of adult smokers. Long-term smoking has been known to lead to anti-oxidant imbalance and inflammation with elevated concentrations of C-reactive protein (CRP), both of which may cause cardiovascular disease. Bo et al. found that treatment with resveratrol reduced systemic inflammation in the airways of patients and decreased CRP released from the liver.^57^

Although resveratrol has been shown to be effective in some clinical trials of various cardiovascular conditions, other studies have reported inconclusive or conflicting results. Zortea et al. found that in schizophrenic patients who are characteristically obese and suffer from metabolic disorders, treatment with resveratrol increased cholesterol and worsened their lipid profiles.^58^ Additionally, Van der Made et al. showed that resveratrol has no effect on HDL (high density lipoprotein) cholesterol levels and on apoA-I concentrations in overweight subjects.^59^ This casts doubt on resveratrol’s ability to reduce the risk of cardiovascular conditions.

Overall, resveratrol has demonstrated positive effects in studies of various cardiovascular conditions, however further research is necessary to verify its effectiveness in human subjects.

### Diabetes

Diabetes affects 422 million people worldwide, with type 2 diabetes comprising 90% of those cases.^60^ Despite increased understanding of this disease and advancements in treatment in recent years, its frequency continues to increase globally, with the WHO projecting that diabetes will be the seventh leading cause of death in 2030. There is much interest in finding a safer, more effective and more affordable therapy to combat this disease. Resveratrol has been shown to improve glycemic control and have antioxidative properties in animal studies, and is therefore being investigated as a promising diabetes therapy. However, because of a limited number of clinical studies, limited sample size and conflicting data, resveratrol’s effectiveness remains unclear. Nonetheless, a systematic review by Hausenblas et al. revealed that significant improvements in multiple cardiometabolic biomarkers and an excellent safety profile support resveratrol as a leading candidate as an adjunct to pharmacological management of type 2 diabetes mellitus. Specifically, statistically significant positive effects were identified for systolic blood pressure, hemoglobin A1c, and creatinine, but not for fasting glucose, homeostatic model assessment of insulin resistance, diastolic blood pressure, insulin, triglycerides, LDL, or HDL cholesterol.^61^

An important area of interest is whether resveratrol can improve glycemic control in humans. Glycated hemoglobin (HbA1c) levels reflect glycemic control and can, therefore, be used as a predictor of the microvascular and macrovascular complications associated with type 2 diabetes. HbA1c levels seem to be determined by postprandial hyperglycemia.^62^ A study by Bhatt et al. showed that daily resveratrol treatment for 3 months decreased HbA1c levels, systolic blood pressure, total cholesterol, and total protein, improving glycemic control.^63^ Fasting blood glucose also decreased, but not significantly. This suggests that resveratrol could be a possible adjuvant for diabetes treatment. However, Thazhath et al. studied two incretin hormones that affect postprandial hyperglycemia: glucose-dependent insulinotropic polypeptide (GIP) and glucagon-like peptide 1 (GLP-1) from the intestine. In healthy people, both hormones stimulate insulin, but in type 2 patients, only GLP-1 can act to stimulate insulin. GLP-1 can also suppress glucagon secretion and energy intake and slow gastric emptying, thereby targeting postprandial hyperglycemia. In rodent models, resveratrol has been shown to upregulate GLP-1 and lower glycemia, but Thazhath et al. found that in human patients, resveratrol had no effect on GLP-1 secretion, glycemic control, gastric emptying, body weight, or energy intake.^62^ As such, resveratrol’s efficacy in improving glycemic control is indeterminate.

Brasnyo et al. also found that resveratrol did not cause any changes in GLP-1 or GIP levels in diabetes patients. However, they did show that resveratrol significantly decreased insulin resistance and blood glucose and delayed glucose peaks after meals.^64^ This may be due to a resveratrol-induced decrease in urinary ortho-tyrosine excretion, a biomarker of oxidative stress. Oxidative status is a promising area of diabetes research because oxygen free radicals are involved in the insulin resistance characteristic of type 2 diabetes. In addition, Brasnyo et al. found that resveratrol activated the Akt insulin signaling pathway by increasing the phosphoAkt:Akt ratio in platelets.^64^

Crandall et al. studied older adults with impaired glucose tolerance (IGT), a major risk factor for diabetes as well as cardiovascular disease. They found that although fasting plasma glucose was unchanged with resveratrol treatment, peak postmeal glucose and 3-h glucose declined. Postmeal insulin fell as well, and insulin sensitivity improved.^65^ Thus, it seems that resveratrol is a promising therapy for insulin resistance.

### Non-alcoholic fatty liver disease

NAFLD is the most common chronic liver disease world-wide, and is an obesity-related disease.^66,67^ The disease is induced by increased levels of triacylglyceride accumulation, which leads to hepatic steatosis, the primary symptom of NAFLD. As implied by its name, patients with NAFLD consume little to no alcohol, yet their livers pathologically resemble those with alcohol-induced liver damage.^66^ Biochemically, NAFLD is characterized by inactivation of AMPK, hepatic lipid accumulation, decreased insulin sensitivity, and inflammation.^66,67^ As resveratrol has been shown to decrease inflammation, activate SIRT1 and mimic effects of caloric restriction, many have predicted that resveratrol could be a potential treatment option for NAFLD.

As resveratrol has only been tested in a small number of clinical trials, there can be no definitive conclusions regarding its effectiveness as a treatment for NAFLD, although certain trends can be observed. A meta-analysis of placebo-controlled clinical trials indicated that resveratrol treatment has negligible effects on attenuating NAFLD, given the small improvement in NAFLD features.^68^ Clinical trials determined the pharmacological effect of resveratrol by measuring the levels of alanine aminotransferase (ALT) and aspartate aminotransferase (AST), two biomarkers that are upregulated in NAFLD, inflammatory markers such as IL-6 and tumor necrosis factor-α (TNF-α), assessing insulin resistance, and characterizing the patients’ lipid profiles.^67,69–71^ Resveratrol was found to have a beneficial effect on patients with NAFLD when a smaller dose was given over a shorter period of time. When patients were treated with a daily dose of 300 mg resveratrol for 3 months, they had decreased levels of ALT and AST, increased lipid metabolism, along with decreased inflammation and glucose levels.^70^ On the other hand, when patients were treated with high doses of resveratrol for longer periods of time, no beneficial effect could be observed. In one study by Chachay et al., patients were treated with 3000 mg resveratrol for 8 weeks, and in another by Heebøll et al., patients were treated with 1500 mg resveratrol for 6 months. In both studies, there was no difference in the levels of ALT and AST, the lipid profiles, or expression of genes related to NAFLD between the resveratrol and the placebo groups.^67,69^

In addition, one study shows that resveratrol may be a beneficial treatment for NAFLD when used as a supplement to lifestyle modifications, including exercise and diet. The patients in this study showed reduced levels of ALT, inflammatory factors such as IL-6 and NF-κB, as well as improved lipid profiles.^71^ All together, these studies seem to show that while resveratrol might be a promising treatment for patients with NAFLD, it might be most effective under certain conditions, and perhaps mostly as a supplement to recommended lifestyle changes. As mentioned above, definitive conclusions cannot be drawn until more clinical trials testing various dosages, durations of treatment, and contexts of treatment have been conducted.

### Obesity

Clinical trials of resveratrol as an inducer of calorie restriction-like effects as a treatment of obesity have produced mixed results. Timmers et al. found that treatment of obese men with 150 mg/day of resveratrol for 30 days resulted in improved cellular and systemic markers of metabolism, such as increased mitochondrial respiration in the muscle and decreased circulating glucose and triglycerides.^72^ Poulsen et al., however, failed to reproduce the findings out of a similar but slightly larger clinical trial, whereby 24 obese men were treated with 500 mg/day of resveratrol for 4 weeks.^73^ The lack of cellular and physiological responses observed in this study contrasts with abundant preclinical evidence, and suggests that future studies of the therapeutic potential of resveratrol should be conducted in obese patients with more pronounced morbidity, such as type 2 diabetes or NAFLD.

## Conclusions

The clinical trials presented in this review show that resveratrol’s therapeutic efficacy depends on several factors. Resveratrol was more effective in certain types of cancer than in others. For example, it seems to epigenetically reduce the expression of certain breast cancer-related genes, but caused severe adverse events specifically in multiple myeloma patients. The vast amount of preclinical data in support of resveratrol’s use as chemopreventive or chemotherapeutic agents warrant further clinical studies. Treatment of patients with AD and stroke was beneficial in all three clinical trials presented, suggesting that resveratrol would be an effective treatment for neurological disorders. However, more clinical trials in this area must be conducted in order to validate this trend. Resveratrol was found to be beneficial for patients with cardiovascular disorders, but perhaps more so in certain demographics than in others, as it was not found to be effective in extremely overweight individuals, and detrimental in schizophrenic patients. In diabetic patients, resveratrol was able to increase insulin sensitivity, decrease blood glucose levels, and positively regulate several other biomarkers associated with diabetes. The effects of resveratrol on NAFLD remain inconclusive, as half of the clinical trials found that resveratrol positively affected NAFLD biomarkers, while the other half observed no changes in those same biomarkers. Similarly, clinical trials of resveratrol in obesity provided conflicting results. Overall, more clinical data are necessary in order to fully understand resveratrol’s therapeutic potential. In addition, future clinical trials should study whether resveratrol is more efficacious in certain patient types. Pharmaceutical efforts should focus on developing a resveratrol derivative with better bioavailability.

### Data availability

Articles cited herein are available via https://www.ncbi.nlm.nih.gov/pubmed/.
